# Negotiating existential conflicts in later life: an empirically informed analysis of gender and age in psychotherapy with older adults

**DOI:** 10.1186/s12939-026-02862-7

**Published:** 2026-04-25

**Authors:** Felix Weibezahl

**Affiliations:** Divison of Ethics in Medicine, Faculty VI Medicine and Health Services Research, Carl von Ossietzky University of Oldenburg, Ammerländer Heerstr. 114-118, 26129 Oldenburg, Germany

**Keywords:** Gendered ageism, Psychotherapy with older adults, Existential psychotherapy, Qualitative methods

## Abstract

**Background:**

This article explores the intersection between age and gender in a psychotherapeutic context with older adults through an existential lens. While awareness of gender- and age-related biases in mental health care is growing, their combined effects and the normative implications for clinical practice remain underexplored. The existential perspective allows psychological symptoms and personal struggles to be understood in relation to the fundamental conditions of human existence, against the backdrop of this intersection and broader structural conditions.

**Methods:**

A secondary qualitative analysis of written psychotherapeutic session protocols from 13 patients aged 60–80 years in psychiatric care was conducted using Kuckartz’s qualitative content analysis. The analysis examined case-based and cross-case patterns to explore potential links between gendered experiences and the negotiation of existential conflicts in later life, thereby generating gender-sensitive hypotheses that may be relevant for clinical practice.

**Results:**

The analysis suggests that structurally gendered conditions, such as economic dependency, the role of professional identity, the amount of involvement in care work, or masculine norms of strength and decisiveness, are linked to how existential conflicts are experienced and negotiated in later life. It offers a basis for hypotheses about how gendered experiences shape encounters with mortality, dependency, and guilt, as well as the resources available for addressing them.

**Conclusions:**

Existential conflicts in later life can be shaped by gendered patterns of experience that influence both the challenges older patients face and the resources they have for engaging with them. An existential perspective complements intersectional approaches by illuminating how structural and fundamentally human dimensions can coalesce in shaping such experiences. This perspective is clinically significant because it draws clinicians’ attention to often overlooked but important patient concerns and supports self-awareness and the development of authentic coping strategies that promote personal growth.

**Trial registration:**

The study was registered with the German Clinical Trials Register (DRKS00023139, 16th November 2020).

## Background

This contribution examines points of contact between the age and gender dimension of engagement with existential conflicts in a psychiatric-psychotherapeutic setting and seeks to elucidate effects of gendered dynamics on the individual experience of existential conflict in later life. While these dynamics may be subtle, they can have tangible consequences for the persons affected. Furthermore, depicting them on an individual level could highlight ethical challenges and provide the basis for their analysis. While the broader issue of gendered ageism provides the backdrop for this inquiry, the central aim of this paper is not to uncover discriminatory practices. Rather, it primarily seeks to explore how the intersection of age and gender manifests within a therapeutic context and how it impacts the care and well-being of older adults in treatment. To do this, the analysis keeps an intersectional perspective in mind, as a frame through which individual differences shaped by a combined effect of multiple social categories are highlighted [1].^1^  

In this article, ‘age’ is understood as encompassing both the later phase of life commonly referred to as old age and the process of ageing itself. This view takes into account that ageing unfolds across biological, psychological, social, and cultural dimensions and is thus an inherently relational phenomenon shaped by bodily changes, psychological adjustment, altered social positions, and cultural narratives [4, 5]. The meanings attached to these transformations differ, not least because the challenges of later life are negotiated within personal histories that are necessarily gendered. Similarly, ‘gender’ is understood as a multidimensional phenomenon, including physical aspects (sex), gender identity, as well as legal and social dimensions, the latter becoming apparent via behaviours shaped by social norms [6].

As the medical discipline attending to the psychopathological consequences of these challenges, psychiatry and psychotherapy offer a unique context for understanding how gender and age intersect in personal experience. Gendered differences in patients’ engagement with themes like loss, identity, and meaning in later life should encourage critical reflection on the clinical frameworks shaping the understanding of these issues. In doing so, the profession would work towards a form of care that avoids reinforcing inequalities and takes diverse experiences into account.

### Psychotherapy with older adults

The principal implication of demographic change for mental health care is that an increased need of psychosocial services for older adults can be anticipated [7]. However, this development also entails evolving perceptions of old age and ageing, which may have implications for the treatment of affective disorders – such as major depression – in this population. There is reason to believe that the prevalence and phenotype of these disorders in old age are underestimated [8].

Psychotherapy is an effective treatment for late-life depression, but older adults rarely receive it due to barriers like a lack of access to specialist care, stigma, and clinician shortages, while antidepressants alone tend to be less effective and are associated with more side effects in this population [9]. For decades, a systematic approach to psychotherapy with older adults was lacking, while therapeutic nihilism shaped by negative age stereotypes prevailed. Today, an increasing number of studies and conceptual frameworks on this topic are available [10]. The major schools of psychotherapy have addressed working with older adults [11–14]. Nevertheless, these approaches tend to emphasize optimization and share a pragmatic perspective, which remains open to ideas from other traditions and typically assumes that working with older adults does not require entirely new psychotherapeutic programs but rather adaptations of existing strategies. While this stance has merit, it may also create blind spots. Notably, important dimensions of experience, such as the existential dimension, are at risk of being overlooked. As a result, the developmental potential of older adults may not be fully appreciated, which in turn risks narrowing the goals of psychiatric-psychotherapeutic practice. Emphasizing the existential dimension of ageing and later life may help to challenge deficit-based conceptions of old age and promote a more comprehensive understanding of affective disorders at this stage of life.

### Ageism and the role of gender in mental health care

Ageism generally refers to discrimination based on age. More specifically, it is the stereotypical construction of older adults, ageing, and old age that is reflected in individual actions, organizations, and cultural values [15]. Specific stereotypes are often conceptualised as ‘views on ageing’ (VoA) [16]. These are often negatively valenced and harmful, not least because they affect individual health [17]. Moreover, it has been shown that ageism can be self-directed and thus negative VoAs are prone to become self-fulfilling prophecies [15]. Consequently, VoAs are a target of this investigation.

In addition, gender does not lose its status as a central category of social inequality in old age. Gender roles and the disparities they may entail play a part in accounting for a lifetime prevalence of depression that is twice as high in women as in men [18]. While there is an increasing awareness of gender- and age-related biases in health care, the nature of their combined effects remains poorly understood [19]. Psychotherapy research has already identified gender as a significant factor in understanding and improving clinical practice [20–22].

An overview of the research literature on mental disorders affirms that gender is a significant social category in mental health care by revealing a range of sex and gender differences in this context.^2^ It has to be taken into account that while the authors of these studies generally recognize that gender differs from biological sex, as it includes social identities, and gender norms, roles, and relationships associated with them, the available data does not allow a clear distinction between the influence of biological, psychological, social, or environmental factors in producing the differences [18].

It is well established that women have a higher lifetime prevalence of depression and anxiety disorders [23, 24]. While it has been argued that sex and gender differences in depression prevalence lessen with increasing age, evidence indicates that older women have an increased risk of major depression, a higher prevalence of anxiety, and report more depressive symptoms, except in the year immediately preceding death, than older men [25–27]. At the same time, the mortality-related risks of mental health issues appear to be greater for older men [18]. In older men, depression carries a higher mortality risk than in older women, and while the severity of the disorder positively correlates with mortality, in older women it stays about the same [25].

One main factor contributing to this greater mortality is suicide. Suicide rates are generally higher in older population groups and especially high for older men [28]. In comparison, suicide rates among women are lower, more stable and more consistent in different parts of the world [18]. To give an example: In Germany in 2024, people aged 60 and older accounted for 57.4% of all suicides in the population [29]. 39.5% were men above 60 and 17.9% were women above 60. This means that more than twice as many men as women in this age group died by suicide. Moreover, an especially high number of unrecorded cases in this age group must be assumed [28].

It is important to note that these sex and gender differences in late-life depression cannot be reduced to measurement artefacts, such as a higher likelihood in women to report symptoms [30]. So far, research has focussed on identifying these quantifiable differences between men and women, and while several biological, psychological, and socio-cultural dynamics have been put forward to account for sex and gender differences in mental health of older adults, there has been less work on the normative dimension of how mental health practitioners should address this issue [18]. The present study employs a qualitative approach to explore the intersection of age and gender, aiming to sensitize clinicians to it and develop insights that could inform recommendations for clinical practice.

### Existential conflict and the existential perspective in psychotherapy

Having established that age and gender intersect as relevant categories in mental health care and that this convergence bears significance for psychotherapeutic treatment, a short definition of ‘existential conflict’ – the phenomenon at the heart of this inquiry – will now be provided.^3^

The term refers to experiences that are essential to being human, because they relate to basic and unchangeable conditions of human experience. Existential conflicts can take different shapes, but they are always associated with finitude. Their central element is that in engaging with them, the contradictory structure of one’s experience of the world becomes apparent. For example, we wish to stay alive but must die eventually, we assign meaning to the things around us while it is not self-evident what this is based on, we strive to be with others but are ultimately separate from everyone else. Dealing with existential conflict is an ambiguous experience, i.e. it can be hard to endure, since it entails suffering and despair, but at the same time holds opportunities for personal growth.

Although the existential dimension of psychological symptoms is not a typical concern for modern psychiatry and psychotherapy, it has received some attention. A diverse body of psychotherapeutic work is categorized under the label ‘existential psychotherapy’, because it draws on existentialist philosophy without necessarily constituting a distinct school of psychotherapy [32]. Emerging from early twentieth-century engagements with existentialist thought, these approaches share philosophical affinities but lack a unified conceptual framework [33].

One of the most influential figures in this tradition is the psychiatrist Irvin Yalom [33]. Like others associated with existential psychotherapy, he identifies a set of fundamental human conditions that give rise to existential conflict or crisis: the ‘ultimate concerns’ of death, freedom, isolation, and meaninglessness [34, p.8f]. Yalom’s account offers a conceptual basis that is helpful for understanding what is meant here by ‘existential conflict’ and ‘existential dynamic’. He defines existential conflict as an intrapsychic struggle ‘that flows from the individual’s confrontation with the givens of existence’ [34, p.8], i.e. with the inescapable conditions of human life. The particular concern in question shapes the form and content of the conflict and its clinical manifestations.

Yalom employs the term ‘existential dynamic’ in reference to the psychodynamic model of mental functioning all psychodynamic therapies are based on [34, p.6]. Here, inner and often unconscious conflicts shape thoughts, emotions, and behaviour. An existential dynamic emerges when the confrontation with a fundamental condition of existence, such as death, gives rise to symptoms, such as anxiety, and the activation of defence mechanisms, such as rationalization. In this sense, the existential dynamic refers to the process connecting existential conflict with its clinical manifestations. This process can be both understood and therapeutically influenced.

Clearly, existential psychotherapy already offers an interpretative framework for understanding psychological symptoms and personal struggles in relation to the fundamental conditions of human existence. However, it remains uncertain to what extent this framework applies to old age. At the same time, approaches to psychotherapy with older adults rarely engage deeply with existential themes, even if they typically acknowledge that ageing involves a heightened confrontation with finitude, which makes existential themes more immediate and is potentially conducive to personal development [13, p.35; 14, 55f; 35]. Some approaches mention only that this confrontation can be challenging for therapists [11, p.93ff].

Its focus on the fundamental conditions of human existence and exploring dynamics between individuals and their world makes an existential perspective well suited to illuminating the ageing-related engagement with finitude in a clinical context. It can shed light on how professionals engage with the irresolvable challenges of human life that are often marginalized by a perspective focused on optimizable functional deficits, even though they can hold clinical relevance and the confrontation with them carries potential for personal development. Additionally, an existential perspective can enrich our understanding of how gender and age intersect to influence individual relations to health, illness, life, or death, going beyond the scope of an intersectional approach, which primarily focuses on socio-cultural power dynamics [36]. It has also been argued that closing this experiential gap, i.e. taking the existential dimension into account, is important for fully understanding psychiatric disorders in ways that can expand on the biopsychosocial model of disease [37].

### Methods

Against this background, a secondary qualitative analysis of psychotherapy protocols with older adults was conducted to explore age- and gender-related dynamics in engagement with existential conflicts. Qualitative methods are particularly appropriate for analysing this underexplored context, because they enable a focus on individual experiences and subjective meanings, and facilitate the examination of areas of overlap between age and gender [38, 39]. Secondary analysis is an approach that is increasingly recognized in qualitative research and involves the re-use of existing empirical material to address new but related research questions [40, 41]. The approach adopted here constitutes a ‘supplementary analysis’ [42], as the research question is essentially a sub-question of that pursued in the primary study. Given that only a short time has elapsed since the primary analysis, that the same researcher is involved, and that access to this specific population and rich material is limited, new data collection was neither necessary nor practical. Moreover, the extensive prior engagement of the researcher with the material rendered the application of a different qualitative method to the same dataset infeasible. Instead, a supplementary analysis of the material enables a focused extension of the primary analysis.

Building on this empirical foundation, the article examines the hypothesis that the negotiation of existential conflict in later life can be meaningfully understood as a gendered experience. It asks whether this perspective deepens our understanding of overlapping vulnerabilities and informs therapeutic approaches within psychiatric and psychotherapeutic contexts. Furthermore, it examines whether the analysis reveals any ethical challenges in the clinical context that may be relevant for practitioners, such as structural or institutional barriers and discriminatory practices that hinder ‘healthy’ ageing. Potential challenges are considered and discussed with regard to possible recommendations for clinical practice.

The analysis is based on written protocols of psychotherapeutic treatment sessions with older adults. These are recorded by the therapist following each session and aim to ensure treatment quality, conserve evidence, and allow for a reconstruction of the treatment process. In absence of binding stylistic guidelines, the form of these protocols varies greatly, but their critical assessment offers valuable insights. The protocols analysed here were originally collected for a study on the significance of existential philosophical concepts in psychotherapy with older adults and were re-evaluated for the purpose of this article. During the primary analysis, gender emerged as a potentially relevant dimension of how existential issues were expressed and negotiated. The present analysis develops this observation further by investigating whether experiences of existential conflict in old age can be understood as gendered and what practical implications follow from this.

In total, the documentation of all sessions in 13 individual cases was used (152 pages, 44784 words). As the sample was purposively selected with regard to the aims of the primary study, participants’ self-designated gender was not assessed. The material does not indicate that any participant’s self-designated gender diverges from the gender attributed to them in the therapist’s documentation. Taking this caveat into account, there were eight female and five male participants, whose age ranged from 60 to 80 years. All had been either inpatients in a psychiatric clinic at one or more times during treatment, had been treated solely in an outpatient setting at the same clinic, or both at different times. The length of their treatment processes varied greatly between three months and almost ten years. Sessions were conducted and documented by psychiatrists or psychologists who were either undergoing psychotherapeutic training or had already completed it. Practitioner continuity was not a requirement for inclusion in the study and could not always be maintained due to treatment length and structural factors of the hospital setting.

All participants consented to the use of their personal data for the purpose of the study. The study protocol was approved by the Research Ethics Committee of the School of Medicine and Health Sciences at the University of Oldenburg (RN 2020 − 138) and the data protection officers of the participating institutions. The study was registered with the German Clinical Trials Register (DRKS00023139, 16th November 2020).

Qualitative content analysis (QCA) according to Kuckartz was used to assess the material [43]. QCA is a well-established method in qualitative social research [44]. Kuckartz’ version allows to account for theoretical preconceptions as well as an explorative approach to the material which serves to develop new hypotheses for future research. Upon receiving consent to participate, all case material was pseudonymized. In some instances, unique elements of cases were altered in ways that preserved the relevant dynamics in order to ensure participants’ privacy. Coding was performed using MAXQDA (2020–2024).

The primary analysis started with a close reading of the material in light of the research question. Relevant passages were marked, and memos were written to record observations. Brief case summaries were produced for each case. In a second step, a set of deductive main categories was developed on the basis of an extensive review of the relevant literature required to address the research question. These categories served as an initial coding grid to structure the material. Building on this, inductive subcategories were developed from the material. The category system thus combined deductive and inductive categories and was continuously refined until theoretical saturation was reached. A coding guide with category definitions and anchor examples was then developed and tested by two independent researchers who coded selected parts of the material. This led to minor revisions of the coding guide, followed by two rounds of coding of the full dataset. Subsequently, thematic summaries were created for each category within each case based on the coded segments. These were condensed into case-based thematic summaries, which formed the basis for cross-case analysis.

The secondary analysis, focusing on the gender dimension of how existential conflict in old age is negotiated in a psychotherapeutic setting, consisted of a re-examination of the entire dataset, including coded segments, thematic and case-based summaries, as well as memos. Existing memos were revisited with a focus on gender aspects that had emerged during the primary analysis, and additional memos were written where relevant. This process identified several cases in which gender-related aspects of experiencing and negotiating existential conflict were particularly pronounced. It is especially the analysis of these cases that informed the development of more gender-sensitive interpretations and working hypotheses.

## Results

The following section presents a selection of cases in which a gender dimension of existential conflict in later life is especially evident. This selective focus serves to illustrate how such patterns emerge from the material and to develop working hypotheses; it does not aim to represent the full range of relevant instances in the dataset.

### Money and mortality

The first featured case is Jane’s, who is 71 years old. The main clinical concern that emerges from the material are various bodily symptoms without any discernible physical cause. The documentation reveals that Jane seems to associate her complaints with the fact that she and her husband have been forced to sell their house:

She associates the start of her hypochondriacal or psychosomatic problems with selling the house; this had been an insult to her. Her mother and others had always said that they couldn‘t afford the house; this has turned out to be true. Moreover, the house represents stability and security. The patient describes strong, diffuse feelings of anxiety which have increased since then. (Jane, Pos. 28)^4^

The quote illustrates that Jane is seen to perceive the forced sale of her home as an insult and a shameful experience, not least because others (like her mother) anticipated this development. Although the precise reason for the sale is not revealed in this excerpt, it can be gathered from other parts of the material in Jane’s case that it is due to her husband’s retirement and the fact that his pension will not suffice to finance the house in the future. At the same time, it emerges that Jane herself has no significant employment history while her husband has been the family’s sole provider. Due to his advanced age, he can no longer ensure the same level of stability as before, which is symbolized by the house. The therapist marks increased insecurity related to this loss: *‘For her the house is related to self-esteem/-worth (“something was taken from me*,* how can I explain this to my friends/acquaintances”) […]’* ^*(Jane, Pos. 35)*^. Most importantly, they establish a link between Jane’s aggravating symptoms and a more intense engagement with death: *‘[…] it becomes clear that her anxiety is quite strongly connected to finitude and mortality (house sale = moving into retirement home*,* giving up autonomy) […]’* ^*(Jane, Pos. 28)*^. Moreover, the material reveals that Jane appears to have always been rather sensitive to her own mortality: *‘She could never accept “that she would one day get something deadly”*,* has always struggled with her own mortality (even as a young girl) […]’* ^*(Jane, Pos.51)*^. This suggests a certain vulnerability that may facilitate a crisis-like experience of existential conflicts in later life.

In Jane’s case, the key gender-related aspect of experiencing existential conflict in old age is her financial dependency on her husband. The material allows for the interpretation that her struggle with the idea of death is shaped by this factor and might unfold differently had circumstances been otherwise. Not least because the confrontation seems forced upon her, given that she cannot change it by financing the house herself.

A deeper look at the material further supports this line of argument and hints at the influence of gender role models in Jane’s case:

[…] she would compare herself to others who have greater income and more luxury, has always done this, especially her father had spoiled her financially, maybe she is cross with her husband that he doesn’t spoil her as much, which he has in fact done up to this point, which is also the reason why they don‘t have any savings to keep the house. (Jane, Pos. 131)

The excerpt suggests that wealth and status have been important for Jane throughout her life and always provided for her by men (first her father, then her husband), until the latter can no longer sustain this state as he becomes older. Correspondingly, the therapist notes their impression that Jane’s primary female role model seems to have favoured this situation by expressing that paid work, and thus providing for oneself, is not appropriate for her: *‘Even when she was working for a short while and liked it*,* her mother had told her that she doesn‘t need to do this.’* ^*(Jane, Pos. 48)*^ The documentation implies that Jane’s upbringing facilitated her having limited trust in herself, a reluctance to step outside her comfort zone, and little responsibility. Presumably, this state of affairs continued throughout her marriage, where she stayed at home while her husband provided financially.

This constellation highlights that gendered aspects of life experience may hinder healthy engagement with existential conflicts in later life, which urge the individual to change and reposition themselves both toward themselves and their world. This can manifest through the impact of gendered aspects of life experience on self-worth and inner stability, the inclination to assume agency over one’s life, or the development of adaptability.

Judging by the material, existential conflicts usually involve multiple interconnected themes. In Jane’s case, the existential theme of death is closely linked to the reevaluation of life decisions and the trajectories they have set in motion, which is most often framed as ‘guilt’, ‘freedom’, or ‘the unlived life’ [45]. The following quote illustrates how engagement with the latter may also be shaped by gender-related factors:

Sometimes she thinks of her first boyfriend and what could have been, had she married him. Her mother had liked him as well; he had been – contrary to her husband – a family man. […] She had also been engaged to her first boyfriend; yet while they had been on vacation she had thrown the ring at his feet during a fight and flown back by herself. Her parents had supported the boyfriend financially during his studies and always kept track of his career as a solicitor. He has a house on Tenerife and a boat. Sometimes her husband (civil servant) says to her “you should have married your lawyer”. (Jane, Pos. 14)

The excerpt focusses on Jane’s perceived continued preoccupation with an early crossroads in her life: Her decision to end her relationship with her first fiancé, who, as the material suggests, ultimately proved to be a better provider of wealth and status than her husband. If one follows the interpretation that she made this decision against her parents’ counsel – ‘Her mother had liked him as well’ – it might also be understood as an instance in which Jane assumed agency in diverging from internalized norms and structures, but in hindsight recognizes her personal responsibility for the consequences of a decision she appears to regret. The exploration of regret about pivotal decisions and developments in her life also extends to aspects such as her choice not to work: *‘She believes that she has missed out on a lot of things which can’t be undone now.’* ^*(Jane, Pos. 48)*^ Yet, it is notable that the presumed desire to have been more self-sufficient, active, or engaged is overshadowed by the idea of having chosen the wrong provider.

### Finding meaning after retirement

The case of Fred, who is 67 years old, serves to illuminate gender-related aspects of engaging with existential conflicts in old age from a male perspective. It can be inferred from the material that Fred retired relatively recently and has sought psychiatric help for the first time during a crisis marked by suicidal ideation. The therapist’s perspective alludes to Fred becoming conscious of his own age in this context. The following excerpt focusses on an everyday situation Fred apparently relates during one session:

He also feels jealous when he “sees other tradesmen”, because they stand “in the prime of their life”. It’s that he was also a tradesman and a successful businessman but now he is asking himself “Who am I anymore? – Nothing!”. (Fred, Pos. 8)^5^

While the material does not reveal whether he reflects on one particular encounter or a repeated experience, one can easily imagine Fred as a patient in a psychiatric clinic walking past men in overalls who are busy repairing a lift or replacing a light. In any case, this sort of encounter seemingly irritates him and leads to feelings of jealousy, presumably because the other men represent a past identity of his. This leaves room for the interpretation that, in coming to terms with no longer being ‘a successful businessman’ and anticipating that others will view him differently as a result, Fred becomes increasingly aware of his own age.

Correspondingly, the therapist’s perspective suggests that Fred’s focus on material life goals and performance does no longer provide the inner stability it used to:

It seems that the patient defines his relationships (especially first marriage) through performance (reaching his life goals: professional independence, buying houses, living by the water, pursuing various hobbies) and derives his emotional well-being from them. (Fred, Pos. 10)

In Fred’s case, the key gender-related aspect of experiencing existential conflict in later life is the emphasis on the threat to his professional role, which serves as the foundation for social recognition and enables ongoing engagement as an active member of society. This becomes even more salient considering the therapist points out several events and developments to Fred that may have contributed to his unstable affective state: his transition into retirement, the deaths of several close family members, caregiving within the family, or an operation he underwent. While the material indicates that Fred does not openly deny the significance of these other factors, the topic that stands out is the loss of his professional identity.

### Uneven resources for engaging with existential conflicts

A comparison between the cases of Fred and Jane raises the possibility that gender may influence not only how existential conflict is experienced, but also what resources are available for addressing it.

When his therapist inquires about Fred’s leisure activities (as a potential resource for coping with his crisis), he talks about his passion: *‘When asked about it the patient talked about his hobby: sailing. This had always given him joy and he had often organized trips (all male group).’* ^*(Fred, Pos. 6)*^ It emerges that Fred pursues a regular activity which involves being in contact with others, i.e. other men, and which he experiences as fulfilling. This is seemingly related to him being made to feel responsible again (as a retiree) and receiving recognition from others: *‘At the weekend he took care of several things (helped a neighbor*,* checked on the boat*,* signed up for slip crew [sic]). Reflects that he felt he had been “given responsibility”.’* ^*(Fred, Pos. 10)*^

In contrast, when Jane’s therapist asks about her plans for the weekend, the answer carries a different tone:

The patient struggles to come up with answers to the question of what she could do at home to not be constantly occupied by her symptoms – lacks leisure activities; doesn‘t want to go out with her husband, doesn‘t have any hobbies except shopping – which she also experiences as unsatisfying. (Jane, Pos. 71)

It emerges that Jane does not pursue any regular activities as part of a social circle where she may take over a specific role and receive recognition from others as a result. On the contrary, her apparent claim that she ‘doesn’t have any hobbies except shopping’ stresses the difference, since it falls in a separate category of social activities than sailing and is here framed as an empty exercise.

In both cases, the material indicates that they are at times being encouraged by their therapists to avoid the existential dimension of their symptoms or are themselves unaware of it. Regardless of how this strategy is judged, the resources available for distraction or for fostering inner stability differ in ways connected to their gendered experience.

### Freedom and responsibility after a partner’s death

The case of Erica, who is 76 years old, reveals a tension between personal freedom and responsibility, that is aggravated by her partner’s death.

The material discloses that she consistently oriented her life around her husband, ceding major life decisions to him, yet remained largely dissatisfied with their relationship: ‘*There had always been severe difficulties in their partnership, which she wants to talk about now’* ^*(Erica, Pos. 46)*^. A closer examination uncovers that Erica’s husband ‘*only ever worked and bought one house after another’* ^*(Erica, Pos.36)*^ while ‘*he never managed to create a real home’* ^*(Erica, Pos. 63)*^. Although it seems possible that, similarly to Jane, Erica was financially dependent on her husband, this cannot be firmly established on the basis of the available material.

The material reveals that Erica takes care of her husband at home after he falls ill and that, more than once, she finds herself in a suicidal crisis that prompts her to seek out psychiatric-psychotherapeutic support. It emerges that during this time, the desire for the freedom to make her own decisions is a key theme: ‘*Sometimes she wishes to find him dead in his bed, so she would still have a few years for a chance to shape her own life.’* ^*(Erica, Pos. 32)*^ Yet, it is also disclosed that after transferring her husband to a nursing home at one point, Erica attempts suicide:

She had not lived her life because she had submitted to her husband’s decisions. […] Caring for her husband seems to have been a „mission“ for the patient, after transferring the husband to a nursing home she attempted suicide. (Erica, Pos. 51)

From an existential viewpoint, this development can be seen as a flight from the possibilities that come sharply into focus at the moment she sets herself ‘free’. The notes reveal her claim that ‘*She has not been true to herself, neglected herself. […] About this she says “I feel cheated”’* ^*(Erica, Pos. 51)*^. Notably, the sense of injustice conveyed in the direct quote implies a tension between the perception of having been prevented from realizing her own possibilities and the realization that their fulfilment has ultimately always been her own responsibility. Following this interpretation, Erica made a choice to relinquish her own potential by deferring all life decisions to her husband. With his death, she loses her guiding figure and is confronted both with the need to make her own decisions and with the realization that she has always been responsible for them: *‘We discussed that she is having problems with making decisions by herself since her husband‘s death.’* ^*(Erica, Pos. 65)*^.

The key gender-related aspect shaping the engagement with existential conflict in Erica’s case is the adoption of a passive role in the creation of a shared life, yielding agency in decision-making. This contrasts well with cases like Fred’s, where men appear as sole providers and decision makers, consistently seeking responsibility and largely excluding their families from these processes. Yet, they may experience difficulties in old age when this sense of responsibility is no longer available to them.

### Dependency and care

Patricia’s case illustrates how existential conflict can center on one’s fundamental relationship with others. The material suggests that, as she becomes conscious of her own age (80), she engages with her vulnerability and the prospect of having to be cared for by others. Preoccupied with a mild physical illness, the idea that she might soon become old, frail and dependent on others appears to gain traction. It can be gathered that Patricia feels rather alone in this situation and perceives her husband as oblivious to the topic of ageing, while she *‘has been the family manager which is increasingly hard for her to do’* ^*(Patricia, Pos. 34)*^.

Ultimately, the documentation reveals a suicide attempt that led to her admission to a psychiatric hospital:

P. recounts a failed suicide attempt and the circumstances that had led her to it. Looking back, she had imagined it to be easier, in light of great pain and feelings of helplessness she had thought that she didn‘t want to be a burden on her children. She talks about caring for her relatives for many years and how she had often looked after others beyond her own limits. (Patricia, Pos. 10–11)

The excerpt conveys the image of a person for whom care work has been an integral part of life, with first-hand experience in caring for family members and an understanding of the sacrifices this entails. An existential perspective highlights how, when faced with the prospect of becoming frail and needing care herself, Patricia considers avoiding engagement with her basic human dependency on others and its impact on relationships through an act she might see as a self-sacrifice.

The central gender-related aspect of experiencing existential conflict in later life that becomes salient in Patricia’s case, is the one-sided assumption of responsibility for care work. This includes persistent feelings of obligation, and internalizing the idea that becoming a burden on others is not permitted or acceptable.

This gender-related aspect emerges even more clearly in light of a contrasting example from the case of Fred:

His mother-in-law‘s care situation, that his wife is quite involved in, burdens him. He finds dealing with suffering generally difficult and also struggles to be around the mother-in-law. At the same time, he doesn‘t want to be involved in decisions about his mother-in-law‘s future, his wife and mother-in-law need to decide this. (Fred, Pos. 4)

The quote reveals a different constellation: Here, what presumably ‘burdens him’ is not the obligation to care for a relative in need, nor the prospect of needing care himself, but rather the necessity of being near a relative who requires care, without being personally responsible for said care. His wife appears to assume the role of caregiver, while Fred, who is reported to find ‘dealing with suffering generally difficult’, is seemingly at liberty to avoid the situation.

Moreover, as in Jane’s case, the therapist records a life-long sensitivity to finitude-associated themes: *‘Being among older (impaired) people and being confronted with the themes of old age and illness had always been stressful for him and gone along with “an uneasy feeling in my stomach”’* ^*(Fred, Pos. 8)*^. While this again suggests a certain vulnerability that may facilitate a crisis-like experience of existential conflicts in later life, it serves only to reinforce the observation that the circumstances under which Fred engages with themes like dependency or suffering appear to differ markedly from those of Patricia. Primarily, because Fred is apparently free to sidestep certain situations of direct confrontation with mortality.

### Gender norms and expectations

Despite the selection so far, not all male cases exemplify stereotypical masculinity. Andrew, who is 77 years old, is confronted with existential themes as well: *‘He cannot explain this new anxiety*,* it seems to be related to the topic of finitude though.’* ^*(Andrew, Pos. 99)*^.

The central issue, which appears to fuel his crisis, is that both his father and grandfather took their own lives at around the same age that he was when he first sought psychiatric help. It emerges that over time Andrew establishes a strong resolve not to follow the path laid out by his male predecessors who *‘[…] had served as a problematic role model’* ^*(Andrew, Pos. 125)*^: *‘He had sworn to himself (and to his father) not to go down that same road.’* ^*(Andrew, Pos. 103)*^ Presumably, rather than keeping his worries to himself, avoiding confrontation, and risking a potentially fatal outcome, Andrew chooses to engage more openly with himself and others and draws strength from doing so. The idea of setting a different example for his own child seems to motivate him greatly.

A closer examination of the material indicates that he has not always conformed to traditional norms of masculinity and that this has been a potential source of difficulty for him:

[…] his main concern at the moment is that his wife might leave him, because she “has had enough of him” (too weak, too sickly), he goes on to report that he had always been “the weak one” and the anxious one (his wife on the other hand the strong one) which had also been the case with his father and grandfather both of whom committed suicide early.’ (Andrew, Pos. 99).

The documentation suggests that Andrew struggled to display stereotypically masculine traits like courage, decisiveness, or leadership throughout his life. In other parts of the material, the therapist records how he *‘[…] talks about his childhood and youth*,* especially about “dares” (crossing a dark tunnel*,* climbing a tree) which he had always been very afraid of.’* ^*(Andrew, Pos. 131)*^ They record Andrew’s painful and persistent preoccupation with such challenges as a child and note that *‘Here*,* a pattern can be observed that is still relevant today.’* ^*(Andrew, Pos. 131)*^.

Ultimately, it emerges that Andrew relies very much on his wife and delegates some stereotypically male tasks to her:

*[…] talks about a hiking trip he had been on with his wife; it becomes clear that he always needs an object that “takes the lead”. When he was once without his wife*,* he immediately got lost and scared. This can be extrapolated to a recurrent motif in his life. (Andrew*,* Pos. 135)*

In Andrew’s case, the key gender-related aspect influencing his engagement with existential conflicts is his ambivalence towards certain masculine gender norms. On the one hand, the internalized expectation of being strong and decisive appears to place him under pressure and contribute to his personal crisis. On the other hand, experiencing the tension between a socially constructed ideal and his inability to adhere to it may also make him more open to seeking help and disclosing personal difficulties.

### Summary

The analysis of the material opens up a perspective on gender as a relevant factor in the negotation of existential conflicts in psychotherapy with older adults. It identifies several gendered patterns that shape engagement with existential issues in later life. These patterns provide a basis for empirically informed hypotheses about the significance of gender in the negotiation of existential conflicts in later life.

A first pattern relates to economic dependency. A lack of control over financial resources appears to bring into focus the fragility of material security and, ultimately, one’s own mortality. While this emerges only in cases of women, a converse pattern in cases of men, for whom their career was of great importance, concerns professional identity as both a stabilising resource and a point of vulnerability. Here, professional identity appears to provide structure, social recognition, and a basis for continued participation in social life after retirement. In contrast, losing a professional identity may destabilize one’s self-image and intensify existential concerns. These gender differences are reflected in processes of life review. While both men and women reflect on past decisions, in cases where dependency is relevant in women’s lives, a thematic focus on unrealized possibilities and a desire for greater self-sufficiency, agency, or having had a professional career is more prominent. Moreover, the resources available for coping with existential distress in the context of ageing, e.g. through leisure activites or social engagement, appear shaped by gendered life experiences.

Another relevant pattern concerns decision-making. The material indicates that a tendency to delegate life decisions to another person, and in this sample it is only women letting men make decisions, may facilitate a confrontation with existential guilt in later life. Moreover, negotiating such existential challenges may be more difficult, because long-term reliance on others for decision-making can make it harder to adapt to autonomy in later life. This is supported when contrasted with cases in which men appear to be the primary decision-makers in their relationships and actively seek responsibility.

A further gendered pattern concerns the assumption of care responsibilities and the relationship to autonomy and dependency this may entail. A tendency to do care work throughout life may be accompanied by internalized norms that frame dependency as problematic which can make it more challenging to come to terms with one’s own vulnerability in later life. At the same time, having few care responsibilites throughout life, which in this sample is only observed in cases of men, may limit encounters with dependency, suffering, and, by extension, one’s own mortality.

A final pattern concerns relationships to male gender norms and role models. Internalized expectations to be strong and decisive may create pressure to engage with existential conflicts, particularly where these involve vulnerability and loss of control. At the same time, tensions between such ideals and the experience of not adhering to them may open up space for reflection and encourage help-seeking, thereby supporting engagement with existential concerns.

## Discussion

The findings on gendered patterns in negotiating existential conflicts in old age resonate with a large body of research on the role of gender as a social category. Several inequalities that disadvantage women have been identified: There is ample evidence for a gender gap in wealth, connected to labour market participation, thus income and ultimately the balance of power in the household [46]. It is also widely appreciated that financial dependence can pose an obstacle to education and employment, as well as exert a negative influence on physical and mental health, not to mention that it is a risk factor for domestic violence [46]. Additionally, financial inequality affects retirement decisions and their outcomes [47]. It is worth noting that men often find it more difficult to adjust to life after losing their professional role following retirement [48]. Furthermore, women spend more time on unpaid childcare and household work and have less free time than men, a pattern described as the free-time gender gap that persists independent of age, income, race, ethnicity, parental status, and marital status [49, 50]. Gender’s influence on factors such as self-worth, inner stability, the inclination to assume agency, and the development of adaptability throughout the life-course has also been widely discussed [51]. Likewise, gendered power imbalances and decision-making patterns within couples, most prominently the ideological consensus around the breadwinner and homemaker model, and recent shifts that seem to ultimately sustain male dominance, have received considerable attention [52]. The results of the present study indicate that these known gender differences can shape individual experiences of existential conflicts. They underline the relevance of factors like economic dependency, professional identity, or autonomy for mental health in later life. Moreover, the findings expand on previous research by showing how these structural inequalities are reflected in individual biographies and may shape how older adults are confronted with existential themes, like mortality, contingency, or guilt, as well as the resources available to them for dealing with these conflicts.

Furthermore, the results suggest that involvement in care work can be associated with an increased likelihood of being confronted with existential themes in later life, whereas a lack of involvement may make it easier to avoid them. This interpretation reflects the gender care gap which captures the unequal distribution of unpaid care work between women and men, with women spending significantly more time each day on such tasks as housework, shopping, childcare, caring for relatives, volunteering, and other unpaid support for households [53, 54]. Another finding suggests that men’s orientation towards social norms of masculinity, which shape how they behave and express their emotions, may influence whether existential conflicts are confronted and what resources are available for engaging with them. Research shows that men’s mental health experiences are strongly influenced by entrenched social norms of masculinity that also affect how they engage with mental health services [55]. Specifically, traditional masculine ideals emphasizing strength, independence, and emotional restraint often discourage help-seeking and increase the risk of depression and suicidal behavior, yet they may also foster resilience and coping in certain contexts [55]. The results of this study make this dynamic more visible and suggest that it may also be relevant for older men as they negotiate existential conflicts.

Taken together, contextualizing the results with research on gender inequalities shows that they not only indicate how gender shapes the negotiation of existential conflict but also highlight the individual and social resources available to address it. Ultimately, the findings support the hypothesis that engagement with existential conflict in later life can sensibly be viewed as a gendered experience. They reveal points of intersection between age- and gender-related dimensions of existential conflict in old age and suggest that gendered power dynamics may shape how such conflicts are internalized and navigated at the individual level. Although the analysis focuses on individual experiences, it brings the relationships between these individuals and their structural conditions into focus. Empirically informed and feminist approaches in bioethics and medical ethics call for this kind of context-sensitive analysis, which aims to make visible how vulnerability and agency are shaped and expressed within specific social and institutional settings [56, 57].

By adopting an existential perspective, the investigation explores an experiential gap that could inform approaches within psychiatric-psychotherapeutic practice. The results illustrate how an existential perspective reveals individuals’ relationships to self and environment on a deeper, more nuanced level that is sensitive to basic human conditions and the contradictory structure of one’s experience of the world. This perspective also supports the development of hypotheses about the impact of social categories on existential conflicts in later life. Thus, it offers a complementary dimension to an intersectional framework limited by its emphasis on socio-cultural power dynamics [36]. The clinical relevance of this view lies in enabling clinicians to uncover underlying concerns that are often overlooked, yet important to patient health and therapeutic outcomes. It conceivably fosters greater self-awareness among clinicians and aids the development of more authentic coping strategies for and with their patients, ultimately facilitating personal growth. Given the responsibility of healthcare professionals to critically evaluate their own biases and professional attitudes to prevent discriminatory practices and promote equitable care, it is reasonable to expect practitioners to reflect on how gendered existential dynamics may shape patients’ experiences in later life. Intersectionality-based frameworks for clinical medicine emphasize this kind of self-reflective practice [58, 59].

Although the material captures the therapists’ perspective, very little is revealed about how they view the influence of age and gender on patients’ situations or the ways in which these factors intersect. Of course, it is conceivable that therapists are aware of the age- and gender-related dynamics highlighted by the findings but do not articulate them in their documentation. This may be especially likely given that note-taking styles vary and are sometimes very reduced. However, the absence of explicit references to these factors in the material should not be interpreted as evidence that they shape clinical practice. On the contrary, it more plausibly suggests that age and gender generally do not play a significant role in clinical reasoning. The fact that sparse indications of explicitly gender- or age-sensitive courses of action and reflection are discernible in the material strengthens the argument that practitioners should be more broadly sensitized to these dynamics to deliver appropriate care. Likewise, the existential dimension of patients’ concerns is rarely acknowledged in the material. One possible explanation is that therapists may unintentionally neglect or avoid this topic because it makes them uncomfortable and relates to something unresolved in themselves, a point also made by Yalom [34, p.13f].

### Negative views on ageing and self-directed ageism

Apart from these general considerations, the analysis also reveals a specific concern for psychiatric-psychotherapeutic practice with older adults: the role of patients’ negative views on ageing in fostering self-directed ageism. As mentioned in the beginning, VoAs are a concept capturing the notions individuals and society hold about old age, older people, and the process of ageing in general [16]. It includes both collective (age stereotypes and societal norms) as well as personal beliefs (self-perceptions of ageing and self-stereotyping). Several cases in the dataset allow for the interpretation that, as patients become increasingly aware of their own age, they are compelled to negotiate a tension between their self-image and their internalized VoAs, which may portray older adults as dependent, frail, or burdensome. In these cases, this negotiation process is accompanied by the emergence of suicidal ideation, suggesting a link between negative VoAs and suicidal thoughts or behaviour. While this connection is tentative, the material conveys a sense of urgency that warrants further investigation of this link. Although a large body of evidence demonstrates the impact that VoAs can have on health, clinicians appear to engage very little with this concept [17, 60]. The absence of any indication in the material that therapists are aware of or actively address negative VoAs emphasizes this gap. This aligns with research showing that negative VoAs influence psychotherapeutic practice [61, 62]. Similarly to therapists’ avoidance of existential topics, this could be linked to being uncomfortable with themes connected to ageing and old age. Thus, the findings suggest that therapists should be made aware of negative VoAs as a form of self-directed discrimination and be encouraged to engage more systematically with VoAs and their clinical implications.

Even though the results allow for a case to be made that mental health practitioners should be aware of gender- and age-related dynamics in how patients navigate existential conflicts, it must also be clarified how such awareness benefits the patient. Because it is not self-evident that imparting knowledge about the conditions contributing to mental distress necessarily leads to its resolution. Rather, the underlying reasoning should be that individualized and equitable care requires psychiatric and psychotherapeutic practice to focus on creating conditions that support personal growth. This includes supporting patients in developing a deeper understanding of their situation, personal history, and their relation to both structural and fundamental human conditions. Sensitivity to these dynamics is necessary to recognize their influence and support patients in navigating them. In this way, individuals may be enabled to engage more authentically with their circumstances and work through the crises they face.

### Limitations and potential of the study

The qualitative, exploratory study design allows for an in-depth analysis of a relatively small number of cases, in accordance with the objective of generating hypotheses and impulses for clinical practice and future research. However, the limited sample size (*n* = 13) does not permit meaningful quantitative conclusions. Additionally, the sample is rather heterogeneous in terms of age, treatment duration, therapist continuity, and therapist’s professional orientation. While the absence of a focus on a specific subgroup (such as the ‘young-old’) could be seen as a limitation, it is compensated for by the potential to uncover themes and challenges pertinent to diverse subgroups within the sample. Since all cases involve a crisis-like experience of ageing due to the study’s selection criteria, examples of ‘uneventful’ ageing cannot be used for comparison.

Moreover, a cohort effect should be considered when interpreting the results. Younger cohorts may hold different views on gender and relate differently to this aspect of human experience; however, they are beyond the scope of studies focusing on older adults.

Notably, the therapists’ gender is not revealed in the examples. From a psychotherapeutic perspective, the gender relationship between patient and therapist carries implications for the treatment process. Although these connections may be interesting, they cannot be adequately studied within this approach for practical reasons (e.g. multiple therapists involved in a case). Thus, the therapists’ gender is omitted so as not to entice the reader to form misleading impressions.

Although sociodemographic data were not systematically collected, the impression emerged that the participants’ socioeconomic backgrounds varied. This can be explained by recruitment taking place in different departments of a large psychiatric hospital that serves as the only provider of psychiatric care in its region and the first point of contact for psychiatric emergencies. Since sociodemographic background affects the likelihood that older adults will seek psychiatric treatment, and study samples in health care research can easily become skewed toward participants with academic backgrounds, future research should take this into account [63, 64].

To appropriately contextualize the findings, it must be considered that the material conveys the therapists’ perspective on the patients’ experience and treatment process; it does not offer direct access to the patients’ perspective. Thus, statements regarding the patients’ experience appear somewhat fragile. However, the material reveals themes, issues, and connections the therapist deems important, presenting them in a way that enables plausible reconstruction suitable for hypothesis generation. It should be taken into account that therapists’ views and assumptions, including their VoAs, likely shape their session notes. As a result, age-specific and existential topics and dynamics may be underreported or overstated. Since the analysis is based exclusively on these notes rather than direct patient accounts, relevant details may be missed or misconstrued, and interpretations may not fully reflect patients’ perspectives on age-related or existential issues. However, as most cases in the dataset, including all those discussed in this article, involved multiple therapists and allowed comparison of session notes over time and across therapists within the same case, this limitation is at least partly mitigated. Consequently, this particular material poses challenges for analysis that should caution against overestimating its informative value. Nevertheless, its potential should not be overlooked: There are no comparable qualitative investigations of psychotherapeutic session protocols available.^6^ Mainly, because these are often considered to be of limited use and difficult to access. Here, the researcher had a relevant professional background. Thus, personal experience within psychiatric and psychotherapeutic settings and in producing documentation of the kind analysed here, has significantly shaped the perspective on the material. While this must be understood as privileged access to the material, it likely made appropriate interpretation possible in the first place. Another important impression emerging from the present study is that it can be advantageous when the researchers have not treated the individuals behind the cases themselves and do not know them personally. In this study, the analysis is based solely on the documentation and not on any personal impressions or experiences beyond what is written. This makes the analysis more transparent to external readers. Overall, the present study demonstrates that this kind of material can be an access point for examining often tacit dimensions of experience that people may not articulate when questioned directly.

QCA according to Kuckartz was chosen for the primary analysis, as its deductive-inductive approach to category development and its strengths in single-case and cross-case analysis were well suited to addressing the research question. Moreover, it enabled the systematic structuring of a type of material that is generally difficult to access. Since the secondary analysis presented in this paper relies heavily on single cases, it could be argued that a different qualitative method, such as objective hermeneutics, would have been more suitable. Nevertheless, re-evaluating the entire dataset using a different method was not considered appropriate, given the researcher’s in-depth prior analysis of the material, which had already informed a structured understanding of the dataset.

Future studies building on these results could test and elaborate on them by incorporating a more direct route to patients’ and therapists’ perspectives. For example, through interviews exploring conscious attitudes and concepts regarding existential conflict and its gender dimension in later life.

## Conclusion

The findings suggest that existential conflicts in later life are shaped by gendered patterns of experience that influence both the challenges that arise and the resources available for addressing them. More specifically, gendered norms around autonomy, care, work, self-reliance, and emotional control can hinder or support authentic engagement with these conflicts. The existential perspective employed complements an intersectional approach to age and gender in a mental health care context by bringing basic human conditions such as finitude into view. It also shows how structural inequalities are reflected in subjective experience, where they can shape one’s relation to self and world. The clinical relevance of this work lies in providing a perspective that can help clinicians notice and work with concerns that are often missed yet relevant for treatment. This perspective makes it easier to recognize how age, gender, and existential concerns intersect in clinically significant ways. In particular, negative VoAs and self-directed ageism appear to be consequential and warrant more systematic consideration in psychiatric-psychotherapeutic practice.

## Data Availability

The datasets analysed during the current study are available from the corresponding author on reasonable request. However, due to the highly sensitive nature of the psychotherapy session protocols, only appropriately pseudonymised versions can be shared.

## Footnotes

1 Intersectionality theory was brought to prominence by feminist scholar Kimberlé Crenshaw in the late 1980s [2, 3]. Since then the framework has been further developed and applied to various fields. Its core tenet is that the social categories shaping individual identities (gender, race, class, age, etc.) coalesce to create structural advantages and disadvantages.

2 This article explores gender differences based on the traditional male-female binary. This is due to the fact that the material was originally collected for a research project on existential conflict in psychotherapy with older adults which did not focus specifically on gender. Accordingly, participants were not asked to self-identify their gender and are categorized here based on the gender recorded in the documentation. Nevertheless, it must be noted that the group of older adults who do not identify with a traditional gender identity has received little attention in mental health care research [18]. Since gender minorities generally experience poorer mental health than the general population, it is likely that disparities and specific challenges will also emerge in this group as they age, highlighting the need for deeper understanding [18].

3 In a previous article based on the same body of material, I employed Karl Jaspers’ philosophical concept ‘limit situation’ to explore existential dynamics in psychotherapy with older adults [31]. For the present analysis, I adopt a more general notion of existential conflict, one that avoids specific philosophical commitments and allows for a clearer focus on the central concern: the influence of gender on existential dynamics in psychotherapy with older adults.

4 All excerpts from the material were translated from the original German by the author. All sensitive names (persons, places, etc.) were replaced by pseudonyms. In some instances, potentially identifying information was altered in ways that preserved relevant patterns while protecting personal identity.

5 A more extensive version of this quote is also discussed in an earlier publication of mine from the perspective of a different research question [31].

6 Levitt describes how qualitative methods have been used in psychotherapy research in the Anglo-American context [65]. There are isolated studies using exact transcripts of therapy sessions and qualitative methods, but none that use the therapists’ own notes as data [66, 67].

## Abbreviations

VoA: View on ageing
QCA: Qualitative content analysis
